# Self-perceptions of aging, physical activity, and depressive symptoms in older community residents with varied neighborhood walkability in Taiwan

**DOI:** 10.1186/s12877-024-05123-w

**Published:** 2024-07-11

**Authors:** Jung-Yu Liao, Yin-Yi Lien, Yung Liao, Yin-Ju Lien

**Affiliations:** 1 Department of Health Promotion and Health Education, National Taiwan Normal University, 162, Heping East Road Section 1, 106, Taipei, Taiwan; 2 Graduate Institute of Sport, Leisure and Hospitality Management, National Taiwan Normal University, 162, Heping East Road Section 1, 106, Taipei, Taiwan; 3 Graduate School of Sport Sciences, Waseda University, 2-579-15 Mikajima, Tokorozawa, 359-1192 Japan

**Keywords:** Physical activity, Mental health, Environment, Self-perceptions of aging

## Abstract

**Background:**

Prior research has identified the mediating effect of physical activity in the relationship between self-perceptions of aging and physical health. However, this impact on mental health is unknown, and the influence of environmental contexts proposed by ecological models in this regard remains largely unexplored. Therefore, this study aimed to investigate the role of physical activity in the relationship between self-perceptions of aging and depressive symptoms in older adults, and compare the impact across four levels of neighborhood walkability.

**Methods:**

A sample of 1,055 community-dwelling older adults aged 65 or above was obtained through random-digit-dialing computer-assisted telephone interviewing. The individual’s neighborhood walkability was calculated using Walk Score®, and categorized into four levels: car-dependent, somewhat walkable, very walkable, and walker’s paradise. Partial least squares structural equation modelling was employed.

**Results:**

We found that more positive self-perceptions of aging were associated with fewer depressive symptoms and a mediation effect of physical activity in this relationship. Among the four levels of neighborhood walkability, the mediation effect of physical activity was only statistically significant in the lowest level (car-dependent). The findings supported our hypotheses regarding the mediating effect of self-perceptions of aging on depressive symptoms via physical activity. Neighborhood walkability might potentially influence the mediating role of physical activity.

**Conclusions:**

This study emphasizes key areas on intervention programs and policy formulation to promote mental health in older adults.

**Supplementary Information:**

The online version contains supplementary material available at 10.1186/s12877-024-05123-w.

## Introduction

Depression is the most prevalent mental disorder among older adults, affecting approximately seven per cent of the world’s older population [1]. Depressive symptoms, regarded as subthreshold depression, can increase the risk of major depressive disorder [2]. Older adults with depressive symptoms are more likely to report poor quality of life [3] whereas less depressive symptom is associated with successful aging [4]. Thus, more attention is being paid to exploring protective factors in relation to depressive symptoms.

Self-perceptions of aging refer to an individual’s evaluation of their aging experience [5], which influences the health of older adults, decreases depressive symptoms, and promotes healthy aging [6]. As suggested by the stereotype embodiment theory [7], older adults who are exposed to negative-age stereotype cues may internalize poor self-perceptions of aging, perceive themselves as older, and display a substantial increase in deterioration, while those with positive self-perceptions of aging may perceive themselves as younger and perform better on both cognitive and physical tasks compared with their counterparts with negative self-perceptions. A Germany study using a survey of adults aged 40–85 years indicated that negative self-perceptions of aging were associated with deterioration in depressive symptoms [8], while an interventional study successfully improved physical activity levels in adults aged 50–82 by addressing negative views on aging and control perceptions [9]. The relation between self-perceptions of aging and physical activity has been suggested which may prevent depressive symptoms among older adults.

The relation between physical activity and mental health and the underlying mechanism have been identified. According to previous literature, physical activity can increase social engagement [10]; this keeps older adults from loneliness and reduces stress, which are strong risk factors for late-life depression [11]. Older adults who engage in physical activity may have less depressive symptom because it can provide a sense of enjoyment, fulfilment, self-efficacy, and social interactions [12], improve the endocannabinoid system function [13], and reduce chronic inflammation [14]. The growing body of literature provides promising support for the significant association between higher physical activity and decreased depressive symptoms in older adults. Thus, physical activity has been regarded as an effective non-pharmaceutical intervention for mitigating depression among older adults [10, 15]. Even during the coronavirus disease 2019 (COVID-19) pandemic, research found that older adults increasing physical activity correlates with reduced depressive symptoms [16].

The relationships between self-perceptions of aging, physical activity, and depressive symptoms remain unclear; however, previous studies have demonstrated the mediating role of physical activity between self-perceptions of aging and other health outcomes (e.g., physical health and quality of life). Cross-sectional studies conducted among Korean and Chinese older adults have found that physical activity partially mediates the relationship between self-perceptions of aging and health outcomes such as self-rated health or health-related quality of life [17, 18]. Longitudinal research on German community-dwelling older adults demonstrated that those with more positive self-perceptions of aging were more likely to engage in physical activity, leading to better self-rated health over time [19]. This finding may also apply to mental health. Therefore, we hypothesize that physical activity mediates the relationship between self-perceptions of aging and depressive symptoms.

To the best of our knowledge, contextual-level factors may influence attitudes and behavior of individuals, as well as their mental health [20], a concept supported by Ecological models [21]. A meta-analysis suggested that environmental contexts were among the neighborhood attributes that might negatively impact depression in older adults [22]. There is a growing number of studies identifying various built environment attributes associated with older adults’ physical activities [23–25]. With the rise of urbanization, there is a greater emphasis on walkability which may be attributed to nearby amenities (e.g., shops and services) and design features that promote physical activity. Neighborhood walkability refers to the extent to which an environment facilitates walking and offers opportunities for older adults to engage in physical activity [26]. Neighborhood walkability is associated with a pleasant physical environment, including well-designed streets, buildings, and convenient transportation access [27–29]. However, previous studies have yielded mixed results on the association between neighborhood walkability and physical activity. While a study in Brazil reported a positive association between neighborhood walkability and moderate-to-vigorous physical activity during leisure time [30], a Canadian study found no significant association between walkability and physical activity volume, intensity, or self-reported walking for transportation [31]. With respect to depressive symptoms, a Japanese study demonstrated that neighborhood walkability was potentially beneficial among older adults living in areas with high population density and proximate local areas [32]. Therefore, further research is necessary to understand the role of neighborhood walkability in physical activity and health among older adults.

Past research has not simultaneously focused on the relationships between self-perceptions of aging, physical activity, and depressive symptoms within neighborhood walkability, and the underlying mechanism remains unclear. Further exploration is necessary to understand the potential existence of conditional effects of the environmental context on the relationships between attitudes, behaviors, and the mental health of older adults—an aspect that has received limited research attention [22]. Therefore, given the current dearth of research on this subject, we hypothesize that the mediating role of physical activity in the relationship between self-perceptions of aging and depressive symptoms may be modulated by distinct levels of neighborhood walkability.

This study had two main objectives overall. First, it aimed to examine the mediation effect of physical activity on the relationship between self-perceptions of aging and depressive symptoms among older adults. Second, we examined the indirect effect of self-perceptions of aging on depressive symptoms through physical activity along with different levels of neighborhood walkability. The findings of this study will provide a new direction for understanding the relationship between self-perceptions of aging, physical activity and mental health among older adults.

## Methods

### Study design and sample

The nationwide cross-sectional data were obtained through a random-digit-dialing computer-assisted telephone interviewing (CATI) survey in Taiwan. The inclusion criteria for this study were community-dwelling older adults aged 65 years and older. The respondents were selected through a stratified random sampling by the geographical stratification with four areas in Taiwan (eastern, southern, western, and northern), and then by their gender (male or female) and age group (i.e., 65 − 74, 75+). The exclusion criteria included that any respondent could not understand or answer questions logically even after receiving an explanation (e.g., those with severe cognitive or mental disabilities). Some respondents with incomplete surveys due to interference (e.g., flicker noise or interruptions) were also excluded from the present study.

All telephone interviews using a standardized and structured questionnaire were administered by well-trained interviewers from a telephone research service company between October 2019 and January 2020. The interviewers had to receive at least 8 h of training, and a checklist was used before the survey to identify the participants’ ability to comprehend and answer the questions. The survey was suspended if a participant could not understand or answer specific questions logically, even after receiving an explanation. To ensure data consistency and quality, all the interviewers conducted telephone interviews daily during the same period, and the entire survey process was supervised and equipped with a monitoring system for listening and observation. A total of 2,352 older adults were interviewed, and the final sample size was 1,068, which included participants who completed the survey without any reward (response rate: 45.4%). No significant difference was found between the final sample and the potential target population (about 3,607,127 older adults) [33] on age, gender, and geographic distributions (*p* > .05), indicating the representation.

After data cleaning, 1,055 participants were considered valid and included in our analysis (excluding the place where the Walk Score® data could not be obtained). Before data collection, ethical approval was obtained from the Institutional Review Board of National Taiwan Normal University (No.: 201712HM012). All participants provided verbal informed consent at the beginning of the phone survey. Other information regarding the sampling and data collection in the present study was described in detail in our previous report [34].

### Measures

#### Self-perceptions of aging

Self-perceptions of aging were measured with the five-item Attitudes toward Own Aging (ATOA) subscale of the Philadelphia Geriatric Center Morale Scale [35]. The ATOA subscale was translated into Mandarin Chinese and then back into English by two bilingual translators and confirmed by a psychologist until the two versions were considered completely interchangeable. Responses were asked to indicate whether they agree (score = 0), neutralize (score = 1) or disagree (score = 2) with the statements. The ATOA subscale has been widely used to measure self-perceptions of aging and has shown satisfactory internal consistency [19]. A sample item was “I have as much pep as I did last year.” We deleted the one item (SPA5) because it did not satisfy the criteria of validity. See details in the results section.

#### Physical activity

Physical activity was measured using the Taiwanese version of the International Physical Activity Questionnaire (IPAQ) and telephone short form [36]. The telephone short form of the IPAQ is suitable and widely used in telephone-based surveys among middle-aged to older adults [37]. It has been shown to have acceptable content validity (0.99) and test-retest reliability (0.80) for monitoring physical activity among older adults [36]. Participants were asked whether they had performed different modes of physical activity (walking, moderate-intensity, and vigorous-intensity activities) in the previous seven days. Additionally, participants indicated the frequency (number of days in the last week) and average duration of each activity per day. The IPAQ score, expressed as “metabolic equivalent (MET)-hours/week” was calculated as the MET level which was multiplied by hours of activity per week. Higher scores indicated a greater level of physical activity in the previous week.

#### Neighborhood walkability

Walk Score® was used to measure the walkability of neighborhood environment or the respondents. Regarding the scoring of Walk Score®, firstly, it is calculated by determining a raw score for each geographic location according to the network distance to nine amenity categories of walking destinations (i.e., grocery stores, restaurants, shopping, coffee shops, bank services, schools, entertainment, bookstores, and park). Then, these raw scores are normalized from 0 to 100, adjusting for the “intersection density” and “block length” around each location [38]. The data sources include Google, Factual, Great Schools, Open Street Map, and other open-source data [38, 39]. The Walk Score® has been validated in America and Asia [39–41], and the validation results in Taiwan are consistent with those obtained in Western countries and Japan [39]. To assess the walkability for each participant, we initially requested them to provide information about their residential neighborhoods. Subsequently, one researcher manually entered each respondent’s residential neighborhood into the Walk Score® website to obtain the walk score. Another researcher replicated the process. The obtained walk scores were identical, and no discrepancies were observed. The official website suggests that it can be classified into four levels (Website: http://www.walkscore.com/methodology.shtml): (1) “car-dependent” (walk score: 0–49), (2) “somewhat walkable” (walk score: 50–69), (3) “very walkable” (walk score: 70–89), and (4) “walker’s paradise” (walk score: 90–100).

### Depressive symptoms

The short version of the Center for Epidemiological Studies-Depression Scale (CES-D) was used to assess the depressive symptoms among older adults. The CESD-5 comprises five items that demonstrate a predictive sensitivity of 0.94 with depression status [42]. It has shown good internal consistency (Cronbach’s α = 0.89; [43]). Participants were asked to indicate the frequency (i.e., never or less than one day, 1–2 days, 3–4 days, and 5–7 days) of statements in the previous week, ranging from 0 (never or less than one day) to 3 (5–7 days). The scores on the CESD-5 ranged from 0 to 15, with higher total scores indicating more severe depressive symptoms. A sample item was “I did not feel like eating; my appetite was poor.”

### Statistical analysis

The mediation (indirect effect) analysis was performed using SmartPLS v4.0 software, with partial least squares structural equation modeling (PLS-SEM) and bootstrapping procedures. The strengths of PLS-SEM include greater statistical power for all sample sizes [44] and greater flexibility in investigating and testing various configurations [45]. PLS-SEM presents an opportunity for exploratory research in which the theory is developed initially or in the early stage [44, 45]. In other words, PLS-SEM is considered more suitable than SEM for a composite-based model.

Before examining the structural model across all four walkability subgroups (car-dependent, somewhat walkable, very walkable, and walker’s paradise), testing the measurement model with indicator loadings that < 0.5 is the first step, which should be eliminated [46] to provide acceptable item reliability. The second step was to examine internal consistency reliability, convergent validity, and discriminant validity. Internal consistency reliability was assessed using Cronbach’s alpha, rho_A, and composite reliability (CR). Rho_A might represent a good compromise because it was an approximately exact measure of construct reliability, which usually lies between Cronbach’s alpha and CR, which are too conservative and too liberal, respectively [47]. The minimum of inter-consistency reliability is acceptable with the value of 0.6 in exploratory research [48]. Convergent validity was assessed using the average variance extracted (AVE). An acceptable AVE is 0.5, but an AVE between 0.4 and 0.5 also showed adequate convergent validity if CR > 0.6 [49]. Discriminant validity was assessed using the heterotrait–monotrait (HTMT) ratio of correlations. The HTMT values did not exceed the value of 0.85 to achieve the requirement of discriminant validity [50].

In the PLS-SEM structural model, the standardized root mean square residual (SRMR) was used as a fit indicator, with a recommended value of 0.08 or lower, indicating a good fit. To assess the direct effect (path coefficients), indirect effect, and total effect in the structural model, the bootstrap procedure used random resampling with replacement from the original data to create pseudo-bootstrap samples (10,000 times was used in the present study), and bias-corrected and accelerated (BCa) bootstrapped confidence intervals (CIs) were calculated to examine the significance of these effects. When the 95 per cent CI does not include zero, an effect differs significantly from zero at *p* < 0.05.

Moreover, we incorporated the participant characteristics (i.e., age, gender, education, and marital status) as covariates. However, the structural models did not achieve an acceptable fit with the SRMR after adjusting for these demographic factors. Consequently, we performed a regression-based path analysis using the PROCESS macro for SPSS, while controlling for the aforementioned covariates. Transformed scores were utilized to address concerns related to the non-normality in the regression-based path analysis [51].

## Results

### Participant characteristics

Overall, 52.9 per cent of the participants had 9 years of education or lower. Regarding marital status, 77.6 per cent were married. Regardless of the walk score, around 36.6 per cent of older adults lived in “car-dependent” locations (Table 1). Comparisons of groups with four levels of neighborhood walkability on these background characteristics were not significant.

### The reliability and validity of constructs in the outer and inner models

As shown in Supplementary Table 1, the results of factor loadings with significant values ranging from 0.52 to 0.83 in the outer models exceeded the recommended value of 0.5, except for SPA5, which had low loading among the four groups and was removed. Cronbach’s alpha was acceptable, and rho_A, CR, and AVE satisfied the recommended criteria, indicating that the proposed measurement models qualified with convergent validity and internal consistency in overall sample and in the four subgroups with different levels of walkability. As shown in Supplementary Table 2, all HTMT values were less than 0.85, indicating that discriminant validity was established.

**Table 1:** Background information of participants

	All (*N* = 1055)		Car-dependent (*n* = 386)		Somewhat walkable (*n* = 153)		Very walkable (*n* = 252)		Walker’s paradise (*n* = 264)		*p*
	*n*	%	*n*	%	*n*	%	*n*	%	*n*	%	
Age (years)	M = 73.17	SD = 5.72	M = 73.22	SD = 5.40	M = 73.71	SD = 6.32	M = 72.88	SD = 5.98	M = 73.07	SD = 5.58	0.546 ^a^
Gender											0.669 ^b^
Male	499	47.3	174	45.1	73	47.7	120	47.6	132	50.0	
Female	556	52.7	212	54.9	80	52.3	132	52.4	132	50.0	
Education											0.577 ^b^
Low	558	52.9	211	54.7	85	55.6	130	51.6	132	50.0	
Medium/ high	497	47.1	175	45.3	68	44.4	122	48.4	132	50.0	
Marital status											0.060 ^b^
Married	819	77.6	287	74.4	114	74.5	200	79.4	218	82.6	
Others	236	22.4	99	25.6	39	25.5	52	20.6	46	17.4	

Note: Education was assessed according to the ISCED (UNESCO, 2013) with three levels (low = lower than 9-year school education; medium = secondary school; high = university admitting degree)

^a^*p*-value was obtained from t-test.

^b^*p*-value was obtained from chi-square test.

### Indirect effects of self-perceptions of aging and depressive symptoms: the mediating role of physical activity

Table 2 presents the correlations and data distributions across all participants and four groups of neighborhood walkability. Significant correlations between self-perception of aging, physical activity, and depressive symptoms were found among all participants, but partial significances were observed across groups. Correlations between self-perceptions of aging and physical activity, as well as between depressive symptoms and physical activity, were not significant in the “very walkable” and “walker’s paradise” groups.

**Fig. 1:**
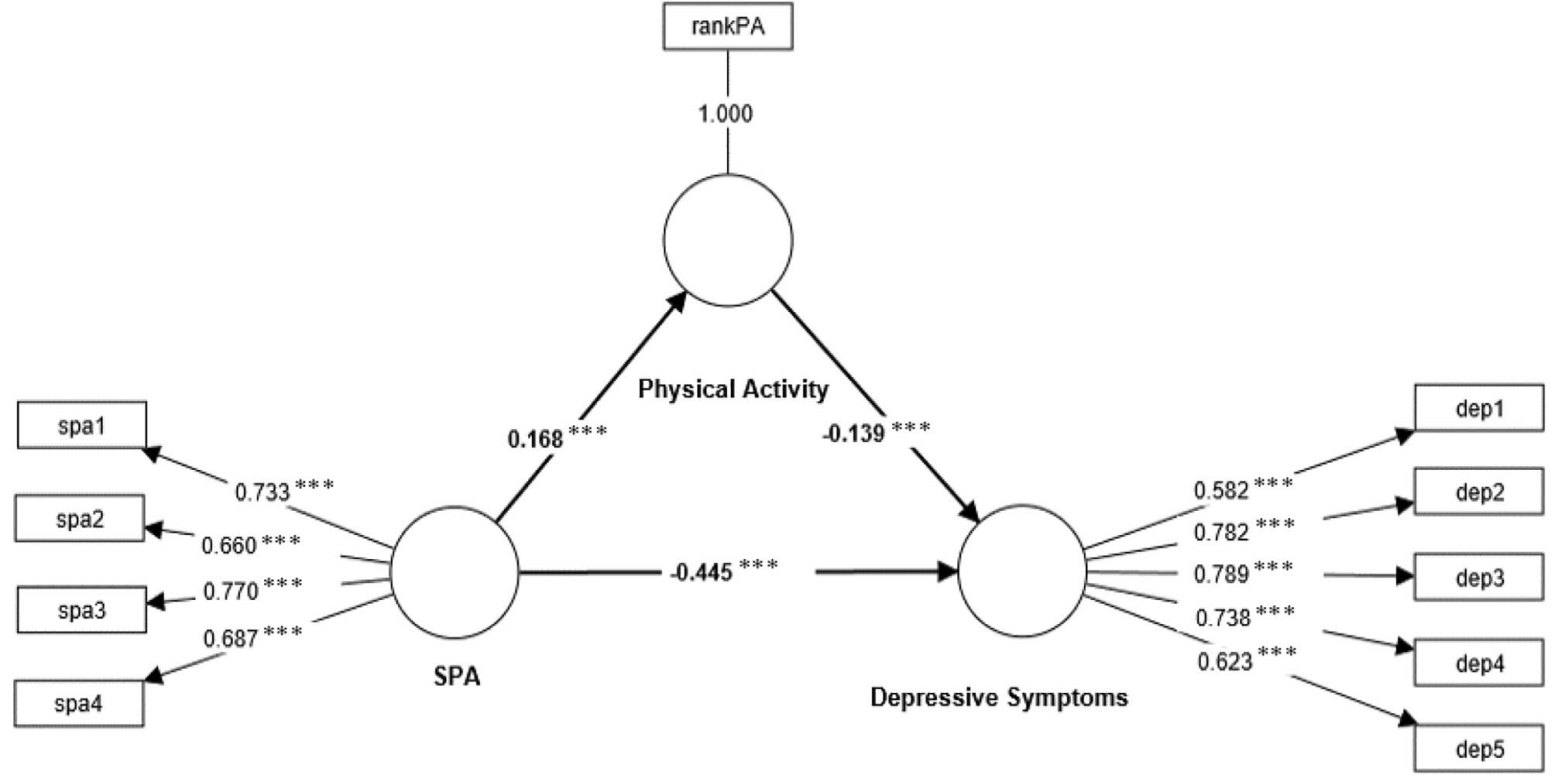
Path coefficients (*p*-values) of constructs among all older adults. ^***^statistically significant at the α = 0.001 level

Figure 1; Table 3 present the results of the PLS-SEM analyses that tested the mediating effects of physical activity on the relationships between self-perceptions of aging and depressive symptoms. The structural model had an acceptable model fit with the SRMR of 0.079. More positive self-perceptions of aging were significantly associated with fewer depressive symptoms (path coefficient = − 0.45, *p* < 0.001). Furthermore, the positive relationship between self-perceptions of aging and physical activity was significant (path coefficient = 0.17, *p* < 0.001). Higher physical activity was associated with fewer depressive symptoms (path coefficient = − 0.14, *p* < 0.001). The indirect effect of self-perceptions of aging on depressive symptoms mediated by physical activity was significant (indirect effect = − 0.02, 95% CI [− 0.04, − 0.01], *p* < 0.001). The BCa CI for the indirect effect of self-perceptions of aging on depressive symptoms through physical activity did not include zero, indicating that the indirect effect was significant. This means that physical activity explained a significant portion of the effect of self-perceptions of aging on depressive symptoms.

**Table 2:** Correlations, means, and standard deviations for study variables across four levels of neighborhood walkability

Neighborhood Walkability	Variables	Depressive Symptoms	SPA	Physical Activity	Mean	SD	Min	Max	Skew	Kurtosis
All	Depressive Symptoms	—	−0.47^***^	-0.11^***^	1.19	2.38	0.00	15.00	2.82	9.04
	SPA		—	0.09^**^	8.63	2.64	4.00	12.00	-0.37	-1.06
	Physical Activity^a^			—	49.75	56.07	0.00	406.00	2.53	9.29
Car-dependent	Depressive Symptoms	—	−0.51^***^	−0.12^*^	1.30	2.42	0.00	15.00	2.61	7.73
	SPA		—	0.10^*^	8.57	2.79	4.00	12.00	-0.36	-1.18
	Physical Activity^a^			—	50.19	51.08	0.00	337.75	1.79	3.93
Somewhat walkable	Depressive Symptoms	—	−0.38^***^	−0.17^*^	1.14	2.36	0.00	15.00	3.08	11.36
	SPA		—	0.18^*^	8.40	2.68	4.00	12.00	-0.30	-1.15
	Physical Activity^a^			—	45.93	52.69	0.00	392.00	2.92	13.55
Very walkable	Depressive Symptoms	—	−0.39^***^	−0.07	1.01	2.20	0.00	15.00	3.22	12.52
	SPA		—	0.03	8.74	2.47	4.00	12.00	-0.28	-1.04
	Physical Activity^a^			—	49.14	52.61	0.00	406.00	2.31	8.83
Walker’s paradise	Depressive Symptoms	—	−0.54^***^	−0.12	1.22	2.49	0.00	14.00	2.72	7.92
	SPA		—	0.09	8.75	2.57	4.00	12.00	-0.46	-0.91
	Physical Activity^a^			—	51.91	67.25	0.00	406.00	2.85	9.80

*SPA* self-perceptions of aging; *SD* standard deviation; *Min* minimum; *Max* maximum. ^a^ Unit is metabolic-equivalent for task (MET)-hr/week.

^*^statistically significant at the α = 0.05 level.

^**^statistically significant at the α = 0.01 level.

^***^statistically significant at the α = 0.001 level.

**Table 3:** Mediated effects of self-perceptions of aging on depressive symptoms through physical activity across four levels of neighborhood walkability

Neighborhood Walkability	Direct effect (95% BCa CI)	Indirect effect (95% BCa CI)	Total effect (95% BCa CI)
All	−0.45^***^ (− 0.49, − 0.40)	−0.02^***^ (− 0.04, − 0.01)	−0.47^***^ (− 0.51, − 0.42)
Car-dependent	−0.48^***^ (− 0.54, − 0.40)	−0.03^*^ (− 0.07, − 0.01)	−0.51^***^ (− 0.56, − 0.44)
Somewhat walkable	−0.37^***^ (− 0.45, − 0.23)	−0.04 (− 0.09, 0.00)	−0.40^***^ (− 0.48, − 0.28)
Very walkable	−0.37^***^ (− 0.45, − 0.23)	−0.01 (− 0.04, 0.00)	−0.38^***^ (− 0.46, − 0.24)
Walker’s paradise	−0.53^***^ (− 0.60, − 0.44)	−0.02 (− 0.05, 0.00)	−0.55^***^ (− 0.62, − 0.46)

*BCa CI* bias-corrected and accelerated (BCa) bootstrap confidence intervals.

^*^statistically significant at the α = 0.05 level.

^**^statistically significant at the α = 0.01 level.

^***^statistically significant at the α = 0.001 level.

### Conditional indirect effects of self-perceptions of aging on depressive symptoms

**Fig. 2:**
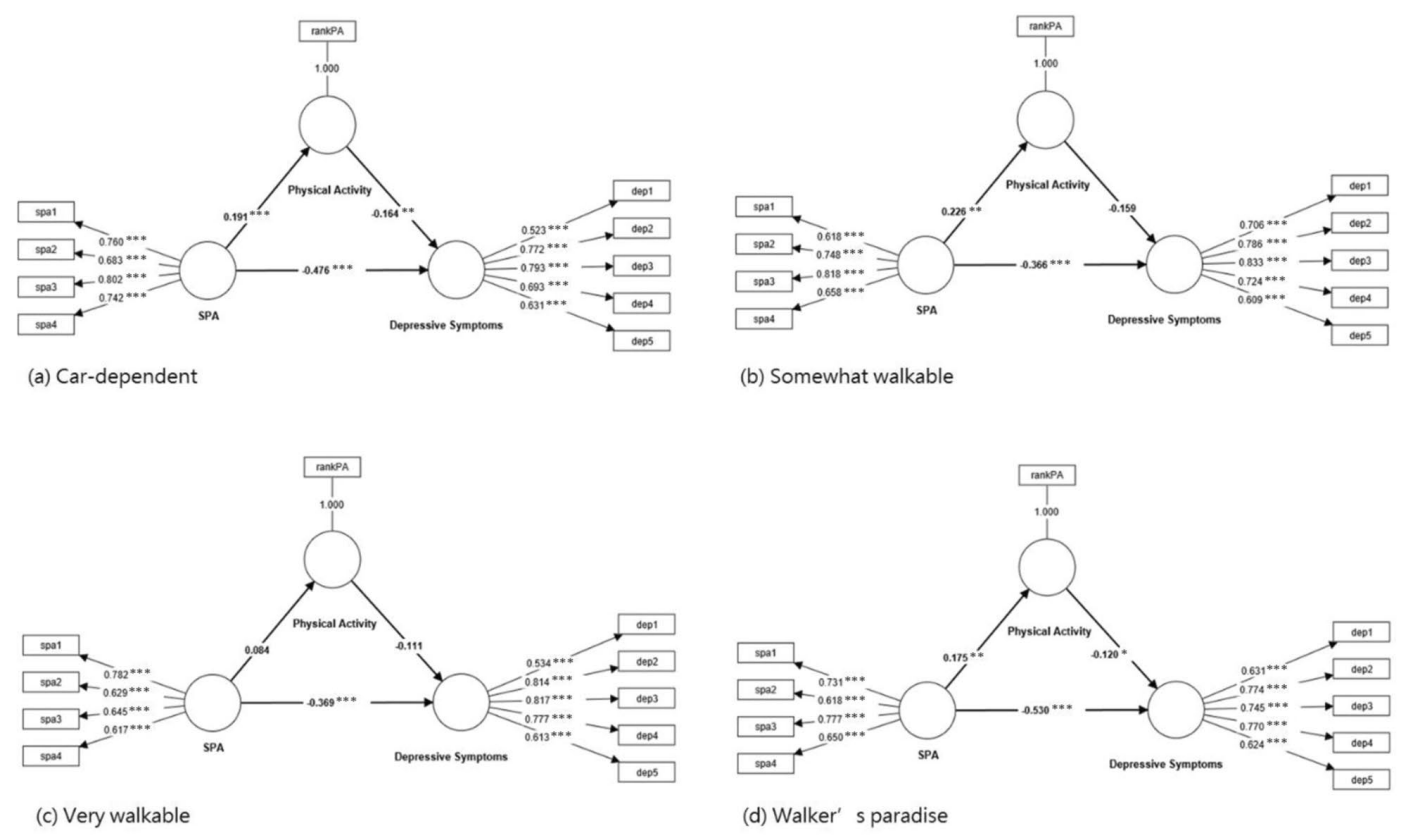
Path coefficients (*p* values) of constructs with four levels of neighborhood walkability. ^*^statistically significant at the α = 0.05 level. ^**^statistically significant at the α = 0.01 level. ^***^statistically significant at the α = 0.001 level

Figure 2 shows the path coefficients of the model among older adults with four walkability levels. The SRMR ranged from 0.064 to 0.072, indicating that the structural models across the four groups had an acceptable model fit. All path coefficients from self-perceptions of aging to depressive symptoms were significant across the four groups. The most negative path coefficient was found in the walker’s-paradise group (path coefficient = − 0.53, *p* < 0.001), followed by the car-dependent group (path coefficient = − 0.48, *p* < 0.001).

The estimations of direct, indirect and total effects are shown in Table 3. The results showed that the indirect effect of self-perceptions of aging on depressive symptoms through physical activity was significant only at the car-depend level (indirect effect = − 0.03, 95% CI [− 0.07, − 0.01]).

### Indirect effects of self-perceptions of aging on depressive symptoms using regression-based path analysis

Comparable results were obtained using regression-based path analysis after adjusting for demographic factors. The estimations of direct, indirect and total effects are shown in Supplementary Table 3. After controlling for demographic factors, the regression-based path analysis results indicated that the indirect effect of self-perceptions of aging on depressive symptoms through physical activity was significant only at the car-depend level (indirect effect = − 0.004, 95% CI [− 0.009, − 0.001]). These findings closely align with those obtained through PLS-SEM.

## Discussion

This innovative study offers Asian data to examine the mediating role of physical activity in the relationship between self-perceptions of aging and depressive symptoms in addition to the conditional effect of neighborhood walkability. The data for this study were collected between October 2019 and January 2020, during a period when Taiwan reported relatively few COVID-19 cases, a trend that continued until 2021. Consequently, our daily lives during the data collection period resembled pre-pandemic conditions. In other words, the findings are interpreted within the context of pre-pandemic normalcy and remain applicable to the post-pandemic period. Our understanding aligns with ecological models, which propose that environmental contexts influence attitudes, behaviors, and health outcomes. In this study, we further explored the conditional effects of environmental contexts on attitude, behavior, and health, which remains unknown. This study addresses a fundamental aspect of this issue. The findings suggest that physical activity partially mediates the relationship between self-perceptions of aging and depressive symptoms. Moreover, we identify a conditional indirect effect of self-perceptions of aging on depressive symptoms through physical activity, indicating variance in the mediation effect based on neighborhood walkability.

In line with our first hypothesis, more positive self-perceptions of aging were associated with fewer depressive symptoms and had a significant indirect effect on depressive symptoms through physical activity. Previous research has found that older adults with more positive self-perceptions of aging tend to engage in physical activity, which can lead to better self-rated health over time [19]. A previous study has reported results consistent with the current study, indicating that the relationship between older adults’ expectations regarding aging and their functional health status is mediated by physical activity [18]. Based on previous literature and our findings in the study, physical activity mediated not only the relationship between self-perceptions of aging and physical health/quality of life but also mental health. Older adults with more positive self-perceptions of aging may be more motivated to stay mobile and active in older age [52]. Individuals who do not expect their health to decline as an inevitable process in old age may be more convinced of their ability to engage in preventing a decline in health, such as through physical activity [53, 54]. More longitudinal data with larger sample size is worthy of being collated to further explore such mediation effects.

Our another finding partially supported the second hypothesis, showing that the mediation effect was only significant at the “car-dependent” level of neighborhood walkability. It suggests a potentially small conditional indirect effect of self-perception of aging on depressive symptoms through physical activity. Previous literature supports the idea that the more walkable the neighborhood where older adults live, the higher their physical activity level, which is inconsistent with the findings of this study. This could be attributed to several factors. One is that older adults who perceive themselves as younger tend to engage in higher levels of moderate-intensity and vigorous-intensity activities rather than walking, especially in ‘car-dependent’ neighborhoods characterized by adjacent rural areas abundant in natural spaces. The stereotype-matching effect may suggest that older adults who perceived themselves as younger are more likely to participate in physical activities [7]. Another reason is that older adults living in walkable or very-walkable environments may have decreased depressive symptoms [32, 55]. Third, walkable neighborhood environments in Taiwan are more crowded and usually more trafficked (i.e., with motorcycles) [56] than in other countries. Some stairs in outdoor environments may enhance the benefits of physical activity but deter older adults from walking [57]. In this environmental context in Taiwan, the mobility of older adults may depend on vehicles instead of walking, even if they live in walkable neighborhoods where most errands can be run on foot. In other words, various scenarios may present when exploring age-friendly and walkable environments for older adults in Asian countries. Other contextual factors should be taken into account. A Japanese study found walkability was not significantly associated with physical activity among older adults but was associated with participation in a recreational program with friends [28]. This finding highlights the importance of considering contextual influences when discussing the pathway from self-perception of aging on depressive symptoms through physical activity. Future research should focus on these factors to develop tailored interventions aimed at promoting active aging and mental health among older adults in diverse environmental contexts.

The findings of the present study carry significant implications, offering actionable insights with global relevance. Improved healthy aging is globally associated with reduced depressive symptoms, suggesting a viable direction for community interventions that focus on enhancing positive self-perceptions of aging and promoting physical activity. A randomized controlled trial study has demonstrated that interventions integrating self-perceptions of aging and exercise have a significant short-term impact on depression, even among older adults with functional limitations [58]. However, the effectiveness of such interventions may vary based on neighborhood walkability levels. The integration of self-perceptions of aging and physical activity becomes especially critical for mental health in poor walkability regions of other countries, as observed in the environmental context of Taiwan. On the other hand, our study further emphasizes the importance of self-perceptions of aging for depressive symptoms among older adults across various neighborhood walkability levels. It serves as a valuable guide for shaping community development strategies globally, highlighting the potential to facilitate healthy aging through the cultivation of positive self-perceptions. In communities, some possible methods for fostering positive self-perceptions involve encouraging the appreciation and celebration of older adulthood, segmenting interventions to focus on the process of healthy aging, and tailoring strategies to be personally and developmentally relevant to older adults of different age groups [6].

### Limitations and future directions

This is the first study to examine the model of stereotype embodiment theory and environmental contexts in depressive symptoms. However, this study has several limitations. First, cross-sectional data limit conclusions regarding causality. Although some biases exist in cross-sectional analyses [59], this study is valuable as a fundamental stage of using a cross-sectional study design and testing the mediated effect of self-perceptions of aging and depressive symptoms via physical activity at different levels of neighborhood walkability before longitudinal data collection. Second, this study with subjective data may have biases, not only in terms of social desirability but also recall biases, although it seems less problematic for the social desirability in telephone surveys than in face-to-face interviews [60]. Third, we did not collect information on the participants’ functional limitations, and physical health, as well as some demographic factors (e.g., race, health status, and income level) and social factors (e.g., family arrangement and the length of residency in the current neighborhoods). These variables could potentially act as confounding factors in research investigating the relationship between neighborhood walkability and mental health. Fourth, we only had Walk Scores for participants’ residential village data, which might cause an inaccurate estimation of walkability. When utilizing the Walk Score® to evaluate walkability in the outer islands of Taiwan, data collection might pose a challenge due to the limited availability of geographic information [39]. We excluded 12 participants living on remote islands, which accounted for 1.14% of the total sample. Subsequently, we conducted a sensitivity analysis by reanalyzing the data, and found consistent results (Supplementary Table 4). Fifth, the IPAQ was employed to measure physical activity in the present study, and it is worth noting that the current IPAQ guidelines might have certain limitations (e.g., it may not fully capture or consider the positive health effects associated with light and sporadic physical activity) [61].

Future research in this field can take several possible directions. Longitudinal data can be collected and used to explore this issue further. One research direction may be to further analyze these associations using walking and moderate-to-vigorous physical activities (excluding walking). The second direction for future research could be to combine subjective and objective measures to assess physical activity and measure functional limitations and physical health as covariates. The third direction for future research could involve integrating a geographic information system (GIS) to assess neighborhood walkability elements, which aiming to enhance our understanding of neighborhood walkability. Moreover, future research should further explore the potential underlying mechanisms of neighborhood walkability in moderating the indirect effect of self-perceptions of aging on depressive symptoms through physical activity. For example, some environmental factors may also influence the relationship between self-perceptions of aging, physical activity, depressive symptoms, and neighborhood walkability, such as neighborhood socioeconomic status [23], degree of urbanization [62], and social environmental factors [63].

## Conclusion

Physical activity plays an important role in the relationship between self-perceptions of aging and depressive symptoms among older adults. This study suggests that intervention programs for decreasing depressive symptoms among older adults should include stimuli for positive self-perceptions of aging, in conjunction with behavioral change programs for an active lifestyle in order to achieve good mental health in later life. In line with ecological theory, our study demonstrates that the individual-level mediation effect may be influenced by contextual-level factors, but the impact is small. The findings of the present study have important implications for the establishment of a walkable neighborhood environment and the development of interventions to promote healthy aging.

### Electronic supplementary material

Below is the link to the electronic supplementary material.

Supplementary Material 1

## Data Availability

All data generated or analysed during this study are included in this published article and its supplementary information files. Adhering to the guidelines outlined by the Local Institutional Review Board, storing data from our ongoing study in a respected repository is not a feasible option.

## Abbreviations

CATI: Computer-Assisted Telephone Interviewing
ATOA: Attitudes Toward Own Aging
IPAQ: International Physical Activity Questionnaire
CES-D: Center for Epidemiological Studies-Depression Scale
PLS-SEM: Partial Least Squares Structural Equation Modeling
CR: Composite Reliability
AVE: Average Variance Extracted
HTMT: Heterotrait–Monotrait
SRMR: Standardized Root Mean Square Residual
BCa: Bias-Corrected and Accelerated (BCa)
CI: Confidence Interval
SPA: Self-Perceptions of Aging
SD: Standard Deviation
Min: Minimum
Max: Maximum
