# Supplementary material for: Self-perceptions of aging, physical activity, and depressive symptoms in older community residents with varied neighborhood walkability in Taiwan
Source: BMC Geriatr. 2024 Jul 11;24:596. doi: 10.1186/s12877-024-05123-w (PMC11242005; doi:10.1186/s12877-024-05123-w)
Supplement: Supplementary file 1 — Supplementary Material 1 [file 12877_2024_5123_MOESM1_ESM.docx]

**Supplementary Table 1** Reliability and convergent validity of self-perceptions of aging, depressive symptoms and physical activity in four levels of neighborhood walkability

| Neighborhood Walkability | Construct | Item^a^ | Factor loadings | Cronbach’s alpha | rho_A | CR | AVE |
| --- | --- | --- | --- | --- | --- | --- | --- |
| All | Depressive symptoms | DEP1 | 0.58 | 0.75 | 0.76 | 0.83 | 0.50 |
| (N=1055) |  | DEP2 | 0.78 |  |  |  |  |
|  |  | DEP3 | 0.79 |  |  |  |  |
|  |  | DEP4 | 0.74 |  |  |  |  |
|  |  | DEP5 | 0.62 |  |  |  |  |
|  | Self-perceptions of aging | SPA1 | 0.73 | 0.68 | 0.69 | 0.81 | 0.51 |
|  |  | SPA2 | 0.66 |  |  |  |  |
|  |  | SPA3 | 0.77 |  |  |  |  |
|  |  | SPA4 | 0.69 |  |  |  |  |
|  |  | SPA5 | -- |  |  |  |  |
|  | Physical activity | rankPA | 1.00 | 1.00 | 1.00 | 1.00 | 1.00 |
| Car-dependent | Depressive symptoms | DEP1 | 0.52 | 0.72 | 0.74 | 0.82 | 0.48 |
| (n=386) |  | DEP2 | 0.77 |  |  |  |  |
|  |  | DEP3 | 0.79 |  |  |  |  |
|  |  | DEP4 | 0.69 |  |  |  |  |
|  |  | DEP5 | 0.63 |  |  |  |  |
|  | Self-perceptions of aging | SPA1 | 0.76 | 0.74 | 0.75 | 0.84 | 0.56 |
|  |  | SPA2 | 0.68 |  |  |  |  |
|  |  | SPA3 | 0.80 |  |  |  |  |
|  |  | SPA4 | 0.74 |  |  |  |  |
|  |  | SPA5 | -- |  |  |  |  |
|  | Physical activity | rankPA | 1.00 | 1.00 | 1.00 | 1.00 | 1.00 |
| Somewhat walkable | Depressive symptoms | DEP1 | 0.71 | 0.78 | 0.79 | 0.85 | 0.54 |
| (n=153) |  | DEP2 | 0.79 |  |  |  |  |
|  |  | DEP3 | 0.83 |  |  |  |  |
|  |  | DEP4 | 0.72 |  |  |  |  |
|  |  | DEP5 | 0.61 |  |  |  |  |
|  | Self-perceptions of aging | SPA1 | 0.62 | 0.68 | 0.72 | 0.81 | 0.51 |
|  |  | SPA2 | 0.75 |  |  |  |  |
|  |  | SPA3 | 0.82 |  |  |  |  |
|  |  | SPA4 | 0.66 |  |  |  |  |
|  |  | SPA5 | -- |  |  |  |  |
|  | Physical activity | rankPA | 1.00 | 1.00 | 1.00 | 1.00 | 1.00 |
| Very walkable | Depressive symptoms | DEP1 | 0.53 | 0.76 | 0.78 | 0.84 | 0.52 |
| (n=252) |  | DEP2 | 0.81 |  |  |  |  |
|  |  | DEP3 | 0.82 |  |  |  |  |
|  |  | DEP4 | 0.78 |  |  |  |  |
|  |  | DEP5 | 0.61 |  |  |  |  |
|  | Self-perceptions of aging | SPA1 | 0.78 | 0.60 | 0.61 | 0.77 | 0.45 |
|  |  | SPA2 | 0.63 |  |  |  |  |
|  |  | SPA3 | 0.64 |  |  |  |  |
|  |  | SPA4 | 0.62 |  |  |  |  |
|  |  | SPA5 | -- |  |  |  |  |
|  | Physical activity | rankPA | 1.00 | 1.00 | 1.00 | 1.00 | 1.00 |
| Walker’s paradise | Depressive symptoms | DEP1 | 0.63 | 0.76 | 0.77 | 0.84 | 0.51 |
| (n=264) |  | DEP2 | 0.77 |  |  |  |  |
|  |  | DEP3 | 0.74 |  |  |  |  |
|  |  | DEP4 | 0.77 |  |  |  |  |
|  |  | DEP5 | 0.62 |  |  |  |  |
|  | Self-perceptions of aging | SPA1 | 0.73 | 0.65 | 0.68 | 0.79 | 0.49 |
|  |  | SPA2 | 0.62 |  |  |  |  |
|  |  | SPA3 | 0.78 |  |  |  |  |
|  |  | SPA4 | 0.65 |  |  |  |  |
|  |  | SPA5 | -- |  |  |  |  |
|  | Physical activity | rankPA | 1.00 | 1.00 | 1.00 | 1.00 | 1.00 |

Note: Physical activity was measured by the MET score. *CR* Composite reliability, *AVE* Average variance extracted

^a^ Named with the abbreviations of responsive constructs: *DEP* Depressive symptoms, *SPA* Self-perceptions of aging, *PA* Physical activity

**Supplementary Table 2** Heterotrait-Monotrait (HTMT) Ratio of correlations for self-perceptions of aging, physical activity, and depressive symptoms in four levels of neighborhood walkability.

| Neighborhood Walkability | Construct | HTMT ratio of correlations |
| --- | --- | --- |
| All | Physical Activity → Depressive Symptoms | 0.25 |
| (N=1055) | SPA → Depressive Symptoms | 0.65 |
|  | SPA → Physical Activity | 0.19 |
| Car-dependent | Physical Activity → Depressive Symptoms | 0.30 |
| (n=386) | SPA → Depressive Symptoms | 0.68 |
|  | SPA → Physical Activity | 0.21 |
| Somewhat walkable | Physical Activity → Depressive Symptoms | 0.27 |
| (n=153) | SPA → Depressive Symptoms | 0.52 |
|  | SPA → Physical Activity | 0.26 |
| Very walkable | Physical Activity → Depressive Symptoms | 0.16 |
| (n=252) | SPA → Depressive Symptoms | 0.54 |
|  | SPA → Physical Activity | 0.11 |
| Walker’s paradise | Physical Activity → Depressive Symptoms | 0.25 |
| (n=264) | SPA → Depressive Symptoms | 0.75 |
|  | SPA → Physical Activity | 0.19 |

*SPA* Self-perceptions of aging

**Supplementary Table 3** Mediated effect of self-perceptions of aging on depressive symptoms through physical activity among older adults by using a regression-based path analysis

| Neighborhood Walkability | Unstandardized estimate (SE) | | | | Indirect effect  (95% BC CI) |
| --- | --- | --- | --- | --- | --- |
|  | a | b | c | c' |  |
| All | 0.13^***^  (0.03) | - 0.02^***^  (0.01) | - 0.11^***^  (0.01) | - 0.10^***^ (0.01) | −0.003^#^  (-0.006, −0.001) |
| Car-dependent | 0.13^**^  (0.05) | - 0.03^**^  (0.01) | - 0.11^***^  (0.01) | -0.11^***^  (0.01) | −0.004^#^  (−0.009, −0.001) |
| Somewhat walkable | 0.16  (0.09) | - 0.04^*^  (0.01) | - 0.09^***^  (0.02) | - 0.09^***^  (0.02) | −0.006  (−0.006, 0.004) |
| Very walkable | 0.09  (0.07) | - 0.02  (0.01) | - 0.09^***^  (0.01) | - 0.09^***^  (0.01) | −0.002  (−0.007, 0.001) |
| Walker’s paradise | 0.11  (0.07) | - 0.02^*^  (0.01) | - 0.12^***^  (0.01) | - 0.12^***^  (0.01) | −0.002  (−0.007, 0.001) |

*BC CI* bias-corrected percentile bootstrap confidence intervals; *SE* standard error

a: the direct effect of self-perceptions of aging on physical activity; b, the direct effect of physical activity on depressive symptoms; c, the total effect of self-perceptions of aging on depressive symptoms; c’, the direct effect of self-perceptions of aging on depressive symptoms

Adjusted for age, gender, education, and marital status.

^#^ There is mediation effect because CI does not include zero.

^*^statistically significant at the α=0.05 level

^**^statistically significant at the α=0.01 level

^***^statistically significant at the α=0.001 level

**Supplementary Table 4** Mediated effects of self-perceptions of aging on depressive symptoms through physical activity across four levels of neighborhood walkability after excluding participants living on remote islands (n = 1043).

| Neighborhood Walkability | Direct effect  (95% BCa CI) | Indirect effect  (95% BCa CI) | Total effect  (95% BCa CI) |
| --- | --- | --- | --- |
| All | −0.45^***^  (−0.49, −0.40) | −0.02^***^  (−0.04, −0.01) | −0.47^***^  (−0.51, −0.42) |
| Car-dependent | −0.48^***^  (− 0.54, −0.40) | −0.03^*^  (−0.07, −0.01) | −0.51^***^  (−0.56, −0.44) |
| Somewhat walkable | −0.39^***^  (−0.45, −0.24) | −0.04  (−0.09, 0.00) | −0.42^***^  (−0.48, −0.28) |
| Very walkable | −0.38^***^  (−0.46, −0.24) | −0.01  (−0.04, 0.00) | −0.39^***^  (−0.46, −0.24) |
| Walker’s paradise | −0.54^***^  (−0.61, −0.45) | −0.02  (−0.05, 0.00) | −0.56^***^  (−0.63, −0.47) |

^*^statistically significant at the α=0.05 level

^**^statistically significant at the α=0.01 level

^***^statistically significant at the α=0.001 level
